# The cost-effectiveness of active surveillance compared to watchful waiting and radical prostatectomy for low risk localised prostate cancer

**DOI:** 10.1186/s12885-017-3522-z

**Published:** 2017-08-08

**Authors:** Chunhuan Lao, Richard Edlin, Paul Rouse, Charis Brown, Michael Holmes, Peter Gilling, Ross Lawrenson

**Affiliations:** 1 National Institute of Demographic and Economic Analysis, The University of Waikato, Level 3 Hockin building, Waikato Hospital, Hamilton, 3240 New Zealand; 2 School of Population Health, The University of Auckland, Auckland, New Zealand; 3 The University of Auckland Business School, The University of Auckland, Auckland, New Zealand; 4 National Institute of Demographic and Economic Analysis, The University of Waikato, Hamilton, New Zealand; 5 Urology Department, Waikato Hospital, Hamilton, New Zealand; 6 Department of Urology, Tauranga Hospital, Tauranga, New Zealand; 7 National Institute of Demographic and Economic Analysis, The University of Waikato, Hamilton, New Zealand

**Keywords:** Active surveillance, Cost-effectiveness, Low risk localised prostate cancer, Radical prostatectomy

## Abstract

**Background:**

Radical prostatectomy is the most common treatment for localised prostate cancer in New Zealand. Active surveillance was introduced to prevent overtreatment and reduce costs while preserving the option of radical prostatectomy. This study aims to evaluate the cost-effectiveness of active surveillance compared to watchful waiting and radical prostatectomy.

**Methods:**

Markov models were constructed to estimate the life-time cost-effectiveness of active surveillance compared to watchful waiting and radical prostatectomy for low risk localised prostate cancer patients aged 45–70 years, using national datasets in New Zealand and published studies including the SPCG-4 study. This study was from the perspective of the Ministry of Health in New Zealand.

**Results:**

Radical prostatectomy is less costly than active surveillance in men aged 45–55 years with low risk localised prostate cancer, but more costly for men aged 60–70 years. Scenario analyses demonstrated significant uncertainty as to the most cost-effective option in all age groups because of the unavailability of good quality of life data for men under active surveillance. Uncertainties around the likelihood of having radical prostatectomy when managed with active surveillance also affect the cost-effectiveness of active surveillance against radical prostatectomy.

**Conclusions:**

Active surveillance is less likely to be cost-effective compared to radical prostatectomy for younger men diagnosed with low risk localised prostate cancer. The cost-effectiveness of active surveillance compared to radical prostatectomy is critically dependent on the ‘trigger’ for radical prostatectomy and the quality of life in men on active surveillance. Research on the latter would be beneficial.

**Electronic supplementary material:**

The online version of this article (doi:10.1186/s12885-017-3522-z) contains supplementary material, which is available to authorized users.

## Background

Radical prostatectomy is the most common treatment for patients diagnosed with localised prostate cancer in New Zealand, [1] though it may cause urinary, sexual and gastrointestinal problems [2]. Active surveillance is considered to be a viable alternative for patients with low risk localised prostate cancer, potentially preventing overtreatment and reducing costs while preserving the option of radical prostatectomy [3]. However, men under active surveillance may suffer from physical complications due to the regular investigations such as biopsies, and issues related to living with cancer, including anxiety and depression [4, 5]. The cumulative risk of a radical prostatectomy increases with time under surveillance.

Watchful waiting is mainly used in patients with a life expectancy less than 10 years, but it was included in two randomised clinical trials to compare with radical prostatectomy [6, 7]. The Scandinavian Prostate Cancer Group Study Number 4 (SPCG-4) showed that men treated with radical prostatectomy had fewer local progression cases, metastatic diseases and cancer-specific deaths than men under watchful waiting after 18 years of follow-up [6]. The Prostate Cancer Intervention versus Observation Trial (PIVOT) found no survival difference between the radical prostatectomy group and the observation group [7]. The inconsistent results between the SPCG-4 study and the PIVOT study might be associated with the different studied cohorts and follow-up time: 5% vs 76% of men identified by screening; 36% vs 43% had low risk cancer; the mean age of 65 years vs 67 years; 45% vs 5% had 15 years follow-up [6–9].

No randomised clinical trial with a follow-up over 10 years has been conducted comparing active surveillance and radical prostatectomy. Two published cost-effectiveness studies [10, 11] comparing active surveillance and radical prostatectomy were based on the PIVOT study [7] where most patients were identified by screening. Given the contradictive evidences of benefits and cost-effectiveness of prostate cancer screening [12–16], a new cost-effectiveness study of active surveillance is needed using data of patients identified clinically. The New Zealand Ministry of Health published guidelines on using active surveillance to manage men with low risk prostate cancer in July 2015 [3]. This study aims to evaluate the cost-effectiveness of active surveillance compared to watchful waiting and radical prostatectomy for men diagnosed with low risk localised prostate cancer in New Zealand.

## Methods

### Ethics

This study was approved by Northern Y (Ref. No. NTY/11/02/019) and Multi-Region Ethics Committees (Ref. No. MEC/11/EXP/044). No inform consent is required for this study.

### Model construction

**Fig. 1:**
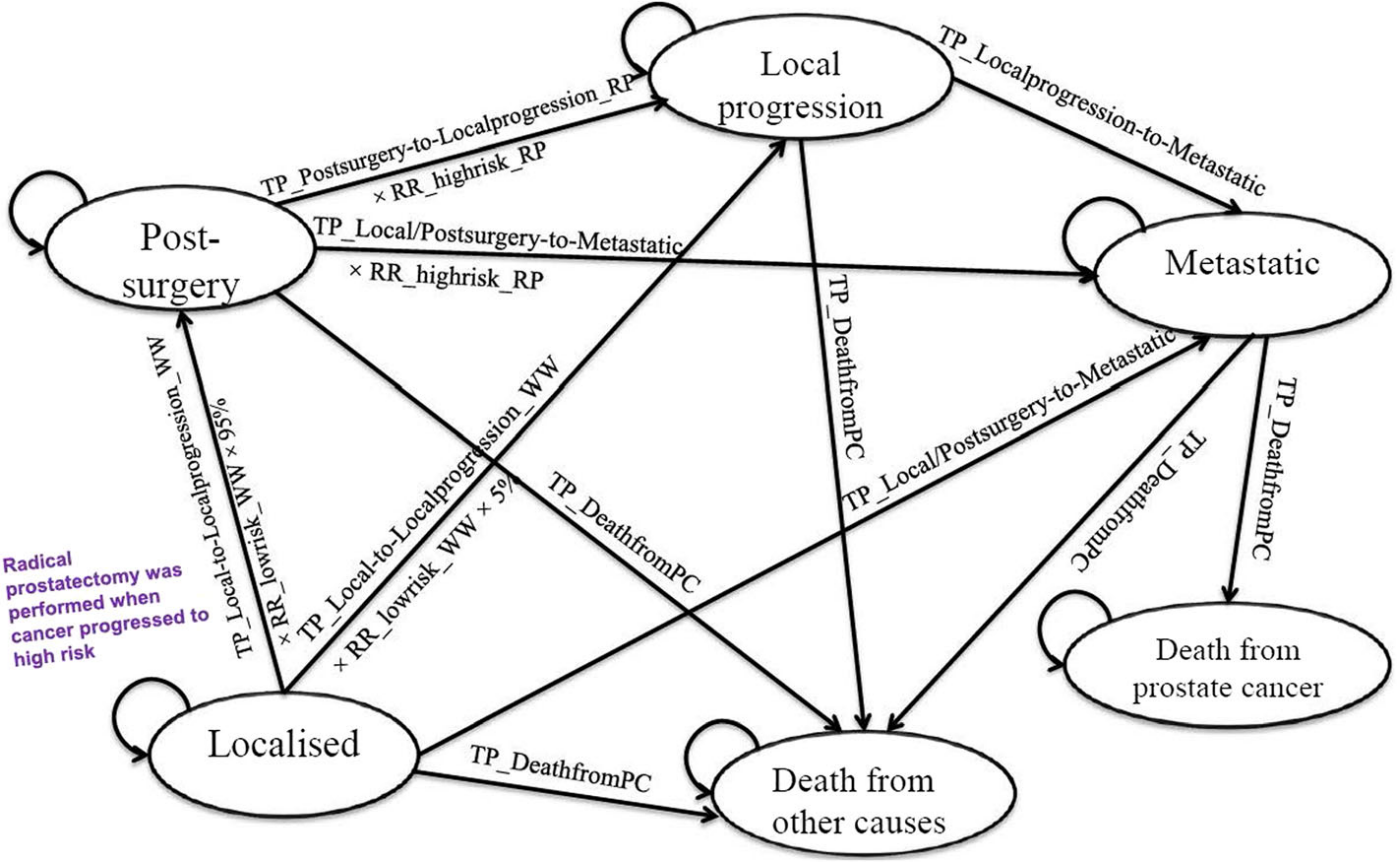
Influence diagram of the Markov model for active surveillance

An economic model was constructed, consisting of three Markov models with microsimulation (radical prostatectomy (Additional file 1: Figure S1), active surveillance (Fig. 1) and watchful waiting (Additional file 1: Figure S2)). The cycle length was 1 year per cycle [17]. The model populations were men diagnosed with low risk localised prostate cancer by the D’Amico risk classification system (biopsy Gleason score ≤ 6, clinical stage T1c-T2a and Prostate-specific antigen (PSA) level ≤ 10 ng/mL) at the ages of 45, 50, 55, 60, 65 and 70 years. The simulations ended when the cohort reached the age of 100.

The health states included ‘Localised’, ‘Post-surgery’, ‘Local progression’, ‘Metastatic’, ‘Death from prostate cancer’ and ‘Death from other causes’ (Fig. 1). In the SPCG-4 study, [6] some men diagnosed with localised prostate cancer in both treatment arms developed metastatic disease in the first year. Therefore, we assumed some metastatic cases were developed directly from ‘Localised’ or ‘Post-surgery’ states. In the active surveillance arm, patients would switch to watchful waiting once they reached 75 years old. When under 75 years old, 95% of them who developed high risk cancer were assumed to be captured and receive radical prostatectomy, and 5% of men were assumed to develop to local progression.

### Transition probabilities

The transition probabilities to ‘Local progression’ from ‘Post-surgery’ in the radical prostatectomy arm (Additional file 1: Figure S3) and from ‘Localised’ in the watchful waiting arm were based on the SPCG-4 study published in 2008 [18]. The transition probabilities to metastatic disease were estimated from the results of the SPCG-4 study published in 2008 and in 2014 [6, 18]. The probability of death from metastatic prostate cancer was estimated based on 276 patients [19–21]. The summarised annual transition probabilities are shown in Table 1.

**Table 1:** Annual transition probabilities in the economic model

Transition probability	Description	Transition probabilities (Mean)	SE			Source
TP_Local-to-Localprogression_WW	From Localised to Local progression in the watchful waiting arm	0.0565	0.0098			[18, 30]
TP_ Postsurgery-to-Localprogression_RP	From Post-surgery to Local progression in the radical prostatectomy arm	0.0152+0.0012T (T: time (years) from radical prostatectomy) Additional file 1: Figure S3	Constant: 0.0026;			[18, 30]
			Slope: 0.0004 Variance-covariance matrix:			
				Slope	Constant	
			Slope	1.80E-07	-9.89E-07	
			Constant	-9.89E-07	6.88E-06	
TP_Local/Postsurgery-to-Metastatic	From Localised or from Post-surgery to metastases	0.0075		0.0010		[6, 30]
TP_Localprogression-to-Metastatic	From Local progression to metastases	0.0800		0.0050		[6, 30]
TP_DeathfromPC	From Metastases to death from prostate cancer	0.3221		0.0115		[19, 21]
TP_Deathfromothercauses	Death from other causes	New Zealand Period Life Tables: 2010–12		-		[31]

The probabilities of progression were estimated from a cohort of men with localised prostate cancer. The relative risks of these transition probabilities for low risk, intermediate risk and high risk cancer (Table 2) compared to the localised cancer cohort were estimated based on the proportions of each risk level cancers in the SPCG-4 cohorts [6, 18] and the relative risks of biochemical recurrence for each risk level cancers [22]. They were estimated by dividing the possibilities of biochemical recurrence for low, intermediate and high risk cancer with the overall possibilities in the two arms in the SPCG-4 study, respectively. The calculation was repeated 100,000 times and Gamma distribution fit the result distribution. The annual likelihood of having radical prostatectomy in the active surveillance arm was assumed to be equal to the transition probability from low risk localised prostate cancer to ‘Local progression’ in the watchful waiting group, and that was 1.6%: TP_Local-to-Localprogression_WW (Table 1) × RR_lowrisk_WW (Table 2).

**Table 2:** Relative risks of cancer progression for low, intermediate and high risk cancer compared to all localised prostate cancer patients in the SPCG-4 study

Relative risk	Risk group	Mean	SE	Distribution
In the radical prostatectomy arm				
RR_lowrisk_WW	Low risk	0.2947	0.0100	Gamma
RR_intermrisk_WW	Intermediate risk	1.0397	0.0347	Gamma
RR_highrisk_WW	High risk	1.9600	0.0655	Gamma
In the watchful waiting arm				
RR_lowrisk_RP	Low risk	0.3006	0.0107	Gamma
RR_intermrisk_RP	Intermediate risk	1.0606	0.0374	Gamma
RR_highrisk_RP	High risk	1.9993	0.0703	Gamma

*RR* relative risk, *RP* radical prostatectomy, *AS* active surveillance, *WW* watchful waiting RR_lowrisk_WW: relative risk of cancer progression for low risk patients compared to localised prostate cancer patients in the watchful waiting arm

### Quality of life

The quality of life data in this model are presented in Table 3. The only quality of life data that specifically addressed active surveillance was from Stewart et al. study [23] (mean value: 0.83). Half men included in that study did not have prostate cancer when the study was conducted. This quality of life value was only used in the scenario analysis (please refer to scenario analyses).

**Table 3:** EQ-5D based quality of life results for patients at different health states

Health states	Treatment	Utility	Disutility	SE	Sources
Post-surgery	Radical prostatectomy	0.900	0.100	0.015	[24]
Localised prostate cancer	Watchful waiting	0.890	0.110	0.013	[24]
	Active surveillance	0.890	0.110	0.013	[24]
Local progression	-	0.820	0.180	0.015	[24]
Metastatic prostate cancer: Not last year in life	-	0.688	0.312	0.019	[32, 33]
Metastatic prostate cancer: final year of life	-	0.551	0.449	0.060	[34]

The quality of life data for active surveillance used in our model was based on a study conducted by Korfage et al. [24]. A quality of life value of 0.89 for men before radical prostatectomy was used as the quality of life for men under active surveillance and a quality of life value of 0.90 after radical prostatectomy was used as the quality of life for men after radical prostatectomy in this model. Our Midland Prostate Cancer Study [21] estimated a similar quality of life value (mean value: 0.88) in 42 men who were diagnosed with localised prostate cancer and had radical prostatectomy.

A utility score of 0.820 for patients who received external beam radiotherapy was used for the utility of patients with local progression, because patients diagnosed with locally advanced prostate cancer are mainly treated with radiotherapy and hormone therapy.

### Costs

This study was from the perspective of the Ministry of Health in New Zealand, and only direct medical costs were considered. The estimated costs excluded goods and services tax (GST) and were valued in 2012/13 New Zealand dollars (NZ$). A 3.5% discount rate was applied to future costs and utilities.

The treatment costs (Table 4) were based on men enrolled in the Midland Prostate Cancer Project and the Metastatic Prostate Cancer Project [20, 21]. Patients with local progression are treated with radiotherapy and hormone therapy which is similar to the treatment pattern for metastatic prostate cancer. The costs were estimated from the National Non-Admitted Patient Collection (NNPAC), National Minimum Dataset (NMDS) and the Pharmaceutical Information Database (PHARMS). These datasets can be linked through patients’ National Health Index (NHI) numbers that is a unique identifier that is assigned to people who use health and disability support services in New Zealand. NNPAC collects national records for outpatient and emergency department events, NMDS contains clinical data for inpatients and day patients, and PHARMS includes all prescribed and dispensed records for subsidised pharmaceuticals.

**Table 4:** Costs of treatment for prostate cancer

Treatment	Treatment year	Age (Years)	Mean	SE	Patients
Localised prostate cancer					
Watchful waiting	First year	All	$323	$193	27
	Subsequent years	All	$0	$0	-
Active surveillance	First year	All	$980	$676	25
	Subsequent years	<75	$812	$651	18
		≥75	$0	$0	-
Radical prostatectomy	First year	All	$13,527	$422	52
	Subsequent years	All	$0	$0	-
Locally advanced and metastatic cancer					
	First year	<80	$8,899	$711	145
	Subsequent years	<80	$6,573	$789	104
	Not last year in life	80+	$3,887	$426	40
	Last year in life	80+	$3,438	$502	75

### Cost-effectiveness analysis

The model construction and data analysis were performed using TreeAge Pro 2015. The model used an outer loop (*n* = 1000) to capture variation in parameter values, with an inner loop microsimulation considering outcomes for a simulated population (*n* = 10,000). The costs and utilities for each simulated man were calculated after each Markov cycle by summing the costs and utilities attached to the related health states and transitions in that cycle. The life-time costs and QALYs (quality-adjusted life years) per simulated man in each treatment arm were estimated by averaging the total costs and utilities of all cycles and applying a half cycle correction to all costs (except the costs of ‘Post-surgery’ in the first year after radical prostatectomy) and utilities.

Uncertainty was assessed in all parameters using appropriate distributions. The probability of progression from ‘Post-surgery’ to ‘Local progression’ is based on two parameters, so the Cholesky Decomposition is used. In all other cases, beta distributions were formed for the other transition probabilities. Gamma distributions were similarly formed to model all disutilities (i.e. the difference between 1 and the relevant utility) and for all cost distributions.

Incremental analysis was performed in terms of incremental cost-effectiveness ratio (ICER) by dividing the incremental life-time costs with the incremental life-time utilities. Cost-effectiveness acceptability curves (CEAC) and frontier plots were also constructed to indicate the likelihood of each treatment being cost-effective under a range of willingness-to-pay values (the amount of money willing to pay for a QALY gained) [25].

### Scenario analyses

Five scenario analyses were conducted. The first scenario analysis used an annual conversion rate of 5% from active surveillance to radical prostatectomy. The 5% conversion rate was used in the cost model built by Corcoran et al. [26]. Hayes et al. used 0.83 (mean value) as the quality of life after active surveillance and 0.80 (mean value) as the quality of life after treatment without complications in their economic model [10, 27]. These quality of life values were used in the second scenario analysis. The third scenario analysis used an alternative set of cost parameters (Additional file 1: Table S1), which were based on the Waikato District Health Board price list. The fourth scenario analysis used the quality of life data in scenario two and the cost parameters in scenario three. The fifth scenario analysis used all the data in the first three scenarios.

## Results

### Cost-effectiveness analysis

Across all five age groups, men in the watchful waiting arm had the lowest life-time costs but also the poorest health outcomes in terms of both life years and QALYs (Table 5). Expected life years were similar between the active surveillance and radical prostatectomy arms, while the number of QALYs was slightly lower for active surveillance. The life-time costs of active surveillance were higher than the costs of radical prostatectomy for men diagnosed aged 45–50, but were lower than the costs of radical prostatectomy for men diagnosed at higher ages.

**Table 5:** Cost per QALY gained for men with low risk localised prostate cancer

Age (years)	Life-time outcome	Watchful waiting	Active surveillance	Radical prostatectomy	Incremental analysis
45	Cost (NZ$)	$15,884	$23,396	$22,316	RP vs WW: $6,432 per QALY;
	Effectiveness (QALYs)	15.43	16.34	16.43	AS was dominated by RP
50	Cost (NZ$)	$14,192	$21,115	$20,991	RP vs WW: $7,906 per QALY;
	Effectiveness (QALYs)	14.49	15.24	15.35	AS was dominated by RP
55	Cost (NZ$)	$12,258	$18,484	$19,612	RP vs WW: $10,358 per QALY; AS was extended dominated by WW and RP (AS vs WW: $10,377 per QALY; RP vs AS: $10,255 per QALY)
	Effectiveness (QALYs)	13.37	13.97	14.08	
60	Cost (NZ$)	$10,113	$15,461	$18,254	AS vs WW: $12,155 per QALY;
	Effectiveness (QALYs)	12.08	12.52	12.65	RP vs AS: $21,485 per QALY
65	Cost (NZ$)	$7,843	$11,998	$16,972	AS vs WW: $14,839 per QALY;
	Effectiveness (QALYs)	10.62	10.90	11.05	RP vs AS: $33,160 per QALY
70	Cost (NZ$)	$5,560	$7,976	$15,821	AS vs WW: $17,257 per QALY;
	Effectiveness (QALYs)	9.03	9.17	9.35	RP vs AS: $43,583 per QALY

*RP* radical prostatectomy, *AS* active surveillance, *WW* watchful waiting

For younger men (aged 45, 50 or 55 years), radical prostatectomy appeared cost-effective compared to watchful waiting with ICERs of NZ$6432 to NZ$10,358 per QALY gained. Active surveillance was dominated (less effective and more costly) by radical prostatectomy for men aged 40–50 and was extended dominated by watchful waiting and radical prostatectomy for men aged 55.

For men aged 60, active surveillance was cost-effective between willingness-to-pay values of around NZ$12,155–21,485 per QALY. At an indicative figure of NZ$30,000 per QALY, radical prostatectomy appeared cost-effective. However, for men aged 65 and 70, the ranges over which active surveillance was cost-effective included this indicative NZ$30,000 per QALY value (NZ$14,839–33,160 per QALY and NZ$17,257–43,583 per QALY). At much lower willingness-to-pay values (e.g. NZ$10,000 per QALY), radical prostatectomy appeared cost-effective for the youngest patients (aged 45 and 50).

The CEACs (Additional file 1: Figures S4 to S9) also provided useful information as to which option is cost-effective at different values of willingness-to-pay. These figures also highlighted that there remained significant uncertainty as to the choice of the most cost-effective option. In all the models up to 60 years of age, there remained at least 30% likelihood that active surveillance was the most cost-effective option at a figure of NZ$30,000 per QALY. Whilst the possibility of radical prostatectomy being cost-effective increased as higher the willingness-to-pay values rise, this option was no more than 65% likely to be cost-effective in any model even at an unrealistic willingness-to-pay of NZ$100,000 per QALY.

### Scenario analyses

The results of five scenario analyses are presented in Table 6. When using the 5% conversion rate (scenario one), the life-time cost of active surveillance increased by 20–36% (aged 70), and the costs of active surveillance were higher than the costs of radical prostatectomy for men aged 45–60 (Additional file 1: Table S2). When using the new quality of life values, the number of QALYs in the active surveillance arm was higher than that in the radical prostatectomy arm in all age groups, and radical prostatectomy was either dominated by active surveillance or extended dominated by active surveillance and watchful waiting. When using new quality of life values, costing values and the 5% conversion rate (scenario five), the ICER of active surveillance compared to watchful waiting increased to NZ$44,090–101,360 per QALY gained. The new costing values (scenario three and four) did not have substantial impact on the results.

**Table 6:** Scenario analysis for men with low risk localised prostate cancer

Age at diagnosis (Years)	ICER (Cost per QALY gained)				Dominance
	AS vs WW	RP vs WW	AS vs RP	RP vs AS	
Scenario one: using the 5% conversion rate					
45	-	$6,441	-	-	AS was dominated by RP
50	-	$7,908	-	-	AS was dominated by RP
55	-	$10,361	-	-	AS was dominated by RP
60	-	$14,021	-	-	AS was dominated by RP
65	-	$21,226	-	-	AS was extended dominated by WW and RP
70	$31,135	-	-	$33,140	-
Scenario two: using new quality of life inputs					
45	$11,060	-	-	-	RP was extended dominated by WW and AS
50	$12,602	-	-	-	RP was extended dominated by WW and AS
55	$14,814	-	-	-	RP was dominated by AS
60	$17,807	-	-	-	RP was dominated by AS and by WW
65	$21,916	-	-	-	RP was dominated by AS and by WW
70	$26,833	-	-	-	RP was dominated by AS and by WW
Scenario three: using new costing inputs					
45	-	$5,324	-	-	AS was dominated by RP
50	-	$6,613	-	-	AS was dominated by RP
55	-	$8,793	-	-	AS was dominated by RP
60	-	$12,332	-	-	AS was extended dominated by WW and RP
65	$15,732	-	-	$24,000	-
70	$19,364	-	-	$35,761	-
Scenario four: using new quality of life inputs and costing inputs					
45	$11,254	-	-	-	RP was extended dominated by WW and AS
50	$12,882	-	-	-	RP was extended dominated by WW and AS
55	$15,248	-	-	-	RP was extended dominated by WW and AS
60	$18,520	-	-	-	RP was dominated by AS and by WW
65	$23,184	-	-	-	RP was dominated by AS and by WW
70	$30,122	-	-	-	RP was dominated by AS and by WW
Scenario five: using new quality of life values, costing values and the 5% conversion rate					
45	$22,904	-	-	-	RP was extended dominated by WW and AS
50	$27,385	-	-	-	RP was extended dominated by WW and AS
55	$33,790	-	-	-	RP was extended dominated by WW and AS
60	$44,090	-	-	-	RP was dominated by WW
65	$59,769	-	-	-	RP was dominated by WW and by AS
70	$101,360	-	-	-	RP was dominated by WW and by AS

*RP* radical prostatectomy, *AS* active surveillance, *WW* watchful waiting

## Discussion

Men in the watchful waiting arm had the lowest life-time costs but also the poorest health outcomes in terms of both life years and QALYs. The model in this study yielded similar numbers of life-years between the active surveillance arm and the radical prostatectomy arm, which was consistent with the evidence that active surveillance and radical prostatectomy have similar effects on the survival of men with low risk localised prostate cancer [3, 28].

The life-time costs of active surveillance were lower than the costs of radical prostatectomy for older men, but were higher for younger men. This likely reflects the fact that the longer a person under active surveillance, the greater the risk of ultimately progressing to surgery and the higher costs. In older men the chance of having surgery is smaller, and active surveillance is a more appropriate tool for them. The cost-effectiveness of active surveillance was dependent on the quality of life for men with localised prostate cancer under different treatment options, and the annual probability of having radical prostatectomy in the active surveillance arm.

The triggers of active treatment in the active surveillance arm remain uncertain and different institutions have their own protocols for both biopsy follow-up and defining need to change from active surveillance to radical prostatectomy [29]. Whether or not these reflected existing protocols, a systematic review including data from seven large active surveillance studies reported that up to one-third of men under active surveillance received definitive treatment after a median follow-up of 2.5 years [28]. It was reported that 27–100% men were treated because of histologic reclassification and 13–48% due to PSA doubling time being less than 3 years, while 7–13% of men were treated without evidence of progression [28].

The model in this study assumed active treatment is triggered only when histological progression of the localised prostate cancer is detected, and an annual conversion rate of 1.6% from active surveillance to radical prostatectomy was used. With an annual conversion rate of 1.6%, life-time costs of active surveillance were lower than the costs of radical prostatectomy for men aged 55–70. This conversion rate is ideal rather than realistic under current practice models.

The cost savings for active surveillance existed because radical prostatectomy either does not occur or is likely to occur a significant time into the future. With higher annual conversion rates, prostatectomies become more likely to occur and to occur sooner. The current surveillance costs incurred can outweigh what is saved by pushing the cost of potential prostatectomies into the future, and in this case the life-time costs of active surveillance can outweigh those of a radical prostatectomy. When using the 5% conversion rate, the life-time costs of active surveillance were higher than the costs of radical prostatectomy for men aged 45–60. A study conducted by Hayes et al. [10] showed that the life-time costs of active surveillance were higher than the costs of radical prostatectomy in men aged 65, using a 9% annual rate of conversion. In reality, the annual probability of converting from active surveillance to active treatment might be over 10% [28].

Quality of life data for men under active surveillance and that for men who received radical prostatectomy appeared critical for the cost-effectiveness of active surveillance compared to radical prostatectomy. Unfortunately, there are no good quality of life data for men under active surveillance. Our Midland Prostate Cancer Study [21] had quality of life data on 42 men with radical prostatectomy (average quality of life:0.88, which is close to the value used in the model) but only 3 men with active surveillance. If the quality of life for men under active surveillance is better than that in the radical prostatectomy arm and men in the active surveillance arm would have radical prostatectomy only when high risk cancer was detected, active surveillance might be cost-effective for men at all age groups. Otherwise it might be only cost-effective for older patients.

One of the strengths of this study is that it synthesized data from internationally recognised studies and local costing and outcome data to provide relevant economic information for decision making in New Zealand. This model was based on data of patients mainly identified clinically instead of by screening, which is different from other cost-effectiveness studies. Variations in different age groups were taken into account, which was an advantage compared to other published studies [10, 11, 26]. The entry criteria for active surveillance in the New Zealand guidelines [3] included a life expectancy of greater than 10 years, but patient’s age was not mentioned. The results of this study might have some impact on these guidelines.

This study has some limitations, including the uncertainties on quality of life for men under active surveillance. The quality of life for men at different ages was assumed to be the same if they had identical treatment. In reality, the quality of life may vary by age even under the same treatment. GP costs were not considered in this study. On average the number of PSA tests per prostate cancer patient per year ordered by GPs was only one, which implied that GPs do not play an important role in the on-going management of prostate cancer patients. The model inputs included the costs of short-term complications (included in the first year costs) caused by radical prostatectomy but not the costs of long-term complications. The long-term complications are mainly managed by GPs, and most of the costs are borne by patients and thus were not considered from the perspective of the Ministry of Health. In the active surveillance arm, radical prostatectomy was assumed to be used when cancer progression was detected. Not taking radiation treatment as definitive treatment into account is a limitation of this study.

## Conclusion

Active surveillance is less likely to be cost-effective compared to radical prostatectomy for younger men diagnosed with low risk localised prostate cancer. The cost-effectiveness of active surveillance compared to radical prostatectomy is critically dependent on the ‘trigger’ for radical prostatectomy and the quality of life in men on active surveillance.

Early or unnecessary trigger of active treatment reduces the cost-effectiveness of active surveillance. If the quality of life for men under observational management was better than that for men having radical prostatectomy, active surveillance was cost-effective compared to radical prostatectomy, but was not cost-effective compared to watchful waiting for older men with a high annual probability of having radical prostatectomy in the active surveillance arm. More research on these specific points may allow a greater certainty when identifying the optimal management for men with low risk prostate cancer.

## Additional file

Additional file 1: Supporting tables and figures for the cost-effectiveness of active surveillance. (DOCX 889 kb)

## Abbreviations

CEAC: Cost-effectiveness acceptability curves
GST: Goods and services tax
ICER: Incremental cost-effectiveness ratio
NHI: National health index
NMDS: National minimum dataset
NNPAC: National non-admitted patient collection
NZ$: New Zealand dollars
PHARMS: Pharmaceutical information database
PIVOT: Prostate cancer intervention versus observation trial
PSA: Prostate-specific antigen
QALY: Quality-adjusted life year
SPCG-4: Scandinavian prostate cancer group study number 4
