# Supplementary material for: The cost-effectiveness of active surveillance compared to watchful waiting and radical prostatectomy for low risk localised prostate cancer
Source: BMC Cancer. 2017 Aug 8;17:529. doi: 10.1186/s12885-017-3522-z (PMC5549326; doi:10.1186/s12885-017-3522-z)
Supplement: Additional file 1: — Supporting tables and figures for the cost-effectiveness of active surveillance. (DOCX 889 kb) [file 12885_2017_3522_MOESM1_ESM.docx]

## Additional file 1

Appendix Table 1. Scenario analysis: annual medical resources used in different treatment arms

| Treatment | Year | Age (years) | Costs | Medical resources |
| --- | --- | --- | --- | --- |
| Watchful waiting | First year | All | NZ$241 | Annual PSA test and one follow-up specialist consultation |
|  | Subsequent years | All | NZ$0 | Referred back to GPs |
|  |  |  |  |  |
| Active surveillance | First year | All | NZ$1,715 | One biopsy, following pathology report, hospitalization due to biopsy complications, 3-monthly PSA tests and two follow-up specialist consultations |
|  | Subsequent years | <75 | NZ$857 | 0.5× (A biopsy, following pathology report, hospitalization due to biopsy complications), 6-monthly PSA tests and one follow-up specialist consultation |
|  |  | ≥75 | NZ$0 |  |
|  |  |  |  |  |
| Radical prostatectomy | First year | All | NZ$12,372 | Radical prostatectomy, hospitalization due to complications and two follow-up specialist consultations |
|  | Subsequent years | All | NZ$0 | Referred back to GP |

Appendix Table 2. Life-time costs per man when using the 5% conversion rate from active surveillance to radical prostatectomy: costs in scenario one

| Age at diagnosis | Watchful waiting | Active surveillance | Radical prostatectomy |
| --- | --- | --- | --- |
| 45 years | $15,880 | $28,028 | $22,321 |
| 50 years | $14,187 | $25,948 | $20,988 |
| 55 years | $12,254 | $23,378 | $19,610 |
| 60 years | $10,119 | $20,206 | $18,251 |
| 65 years | $7,835 | $16,174 | $16,962 |
| 70 years | $5,557 | $10,850 | $15,821 |


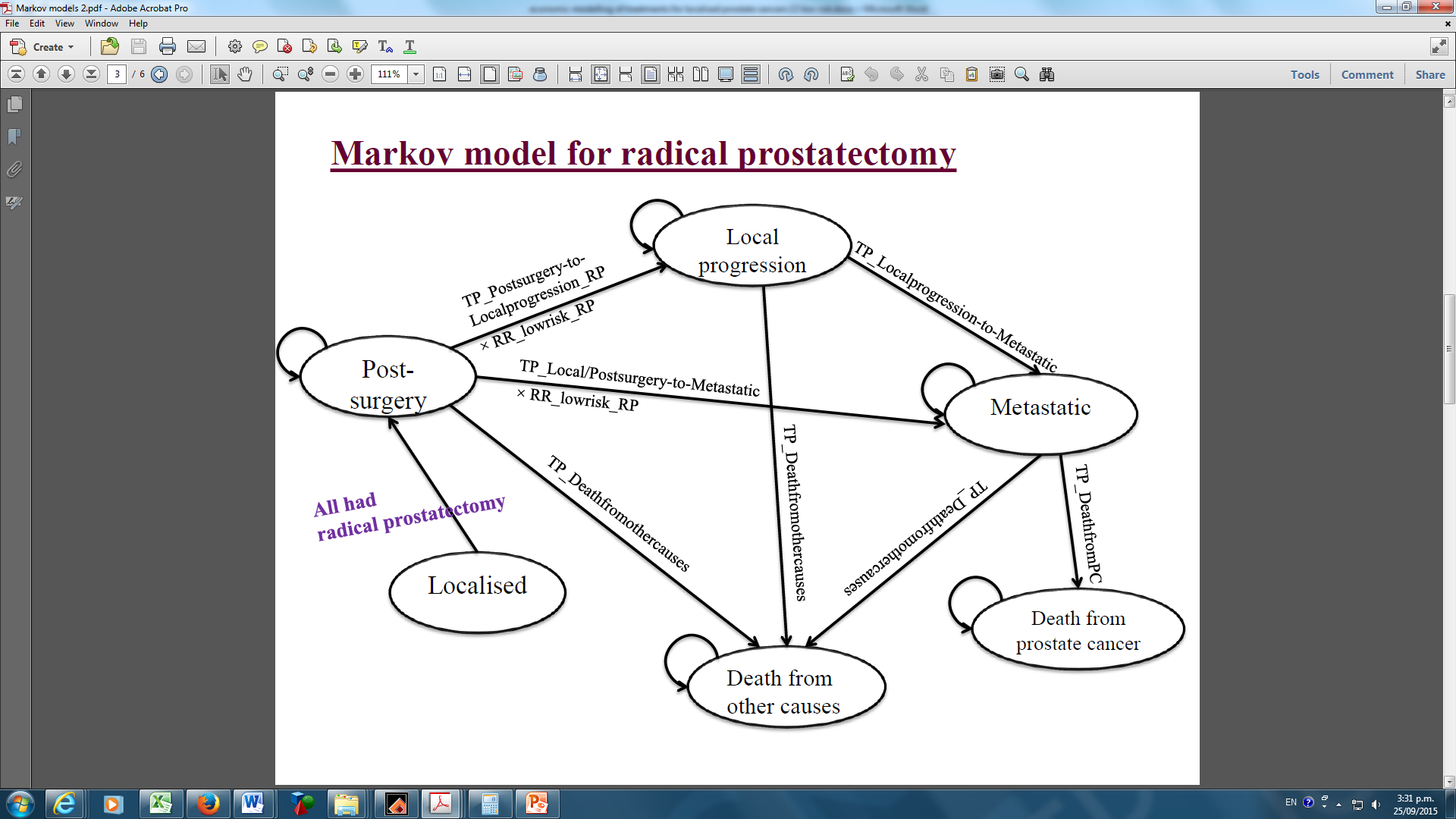


Appendix Figure 1. Influence diagram of the Markov model for radical prostatectomy


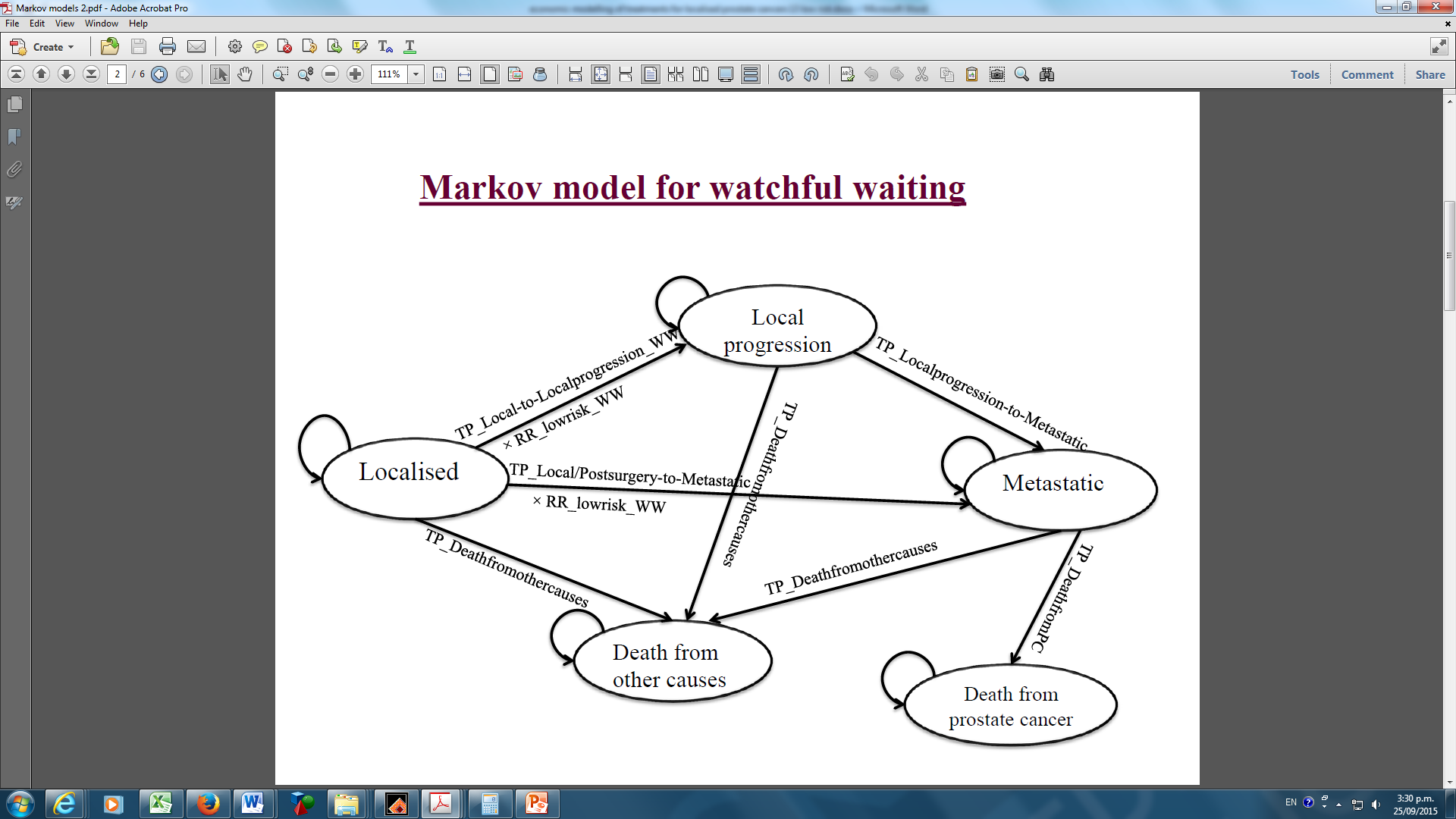


Appendix Figure 2. Influence diagram of the Markov model for watchful waiting


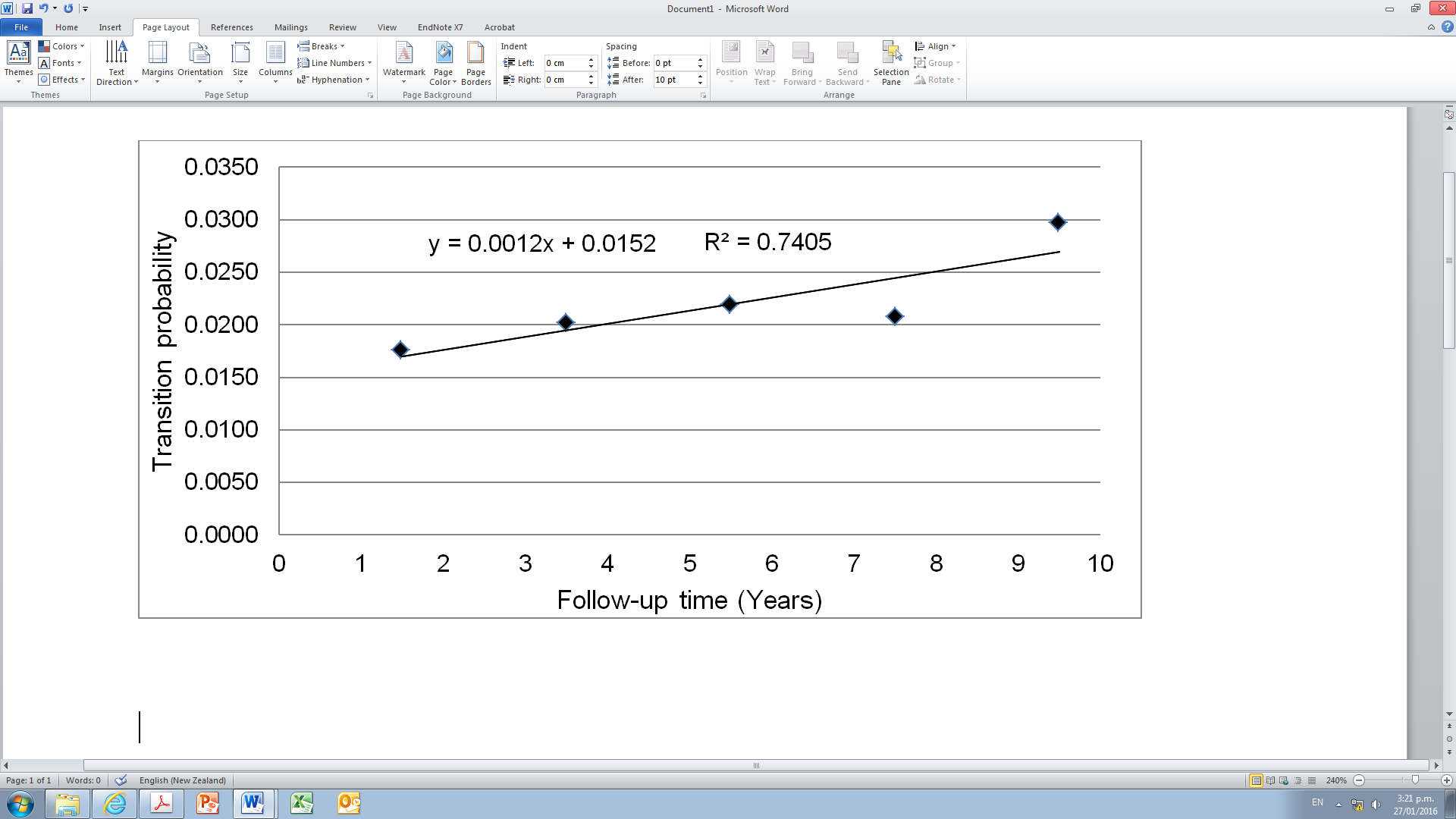


Appendix Figure 3. Correlation between follow-up time and transition probability to ‘Local progression’ from ‘Post-surgery’ in the radical prostatectomy arm in the SPCG-4 study

The transition probabilities to ‘Local progression’ from ‘Localised’ or from ‘Post-surgery’ were estimated using similar method as in the study published by Guyot et al.[35] The rates of local progression were first estimated from the digitalized cumulative hazard of local progression in the SPCG-4 study[18] and were then converted into transition probabilities: tp= 1-EXP(-r*t); tp: transition probability; r: rate; t: time unit.[36] The correlation between follow-up time and transition probability was strong (Appendix Figure 3**:** R^2^=0.7405) in the radical prostatectomy arm, but was much weaker (R^2^=0.1750) in the watchful waiting arm. Therefore, a time dependent annual transition probability from ‘Post-surgery’ to ‘Local progression’ was used in the radical prostatectomy arm, and a constant annual transition probability was estimated in the watchful waiting arm.

Appendix Figure 4. CEAC: men with low risk localised prostate cancer aged 45 years

Appendix Figure 5. CEAC: men with low risk localised prostate cancer aged 50 years

Appendix Figure 6. CEAC: men with low risk localised prostate cancer aged 55 years

Appendix Figure 7. CEAC: men with low risk localised prostate cancer aged 60 years

Appendix Figure 8. CEAC: men with low risk localised prostate cancer aged 65 years

Appendix Figure 9. CEAC: men with low risk localised prostate cancer aged 70 years

References

[35] Guyot, P., Ades, A.E., Ouwens, M. & Welton, N. Enhanced secondary analysis of survival data: Reconstructing the data from published Kaplan-Meier survival curves. [*BMC Med Res Methodol*](http://www.ncbi.nlm.nih.gov/pubmed/22297116) 2012;12:9.

[36] Fleurence, R.L. & Hollenbeak, C.S. Rates and probabilities in economic modelling: Transformation, translation and appropriate application. [*Pharmacoeconomics*](http://www.ncbi.nlm.nih.gov/pubmed/17192114) 2007; 25(1):3-6.
